# Supplementary material for: S2Tag, a novel affinity tag for the capture and immobilization of coiled-coil proteins: Application to the study of human β-cardiac myosin
Source: J Biol Chem. 2025 Sep 29;301(11):110776. doi: 10.1016/j.jbc.2025.110776 (PMC12597268; doi:10.1016/j.jbc.2025.110776)
Supplement: Figures S1-S4 [file mmc1.docx]

**Supporting Information**

**S2Tag, a novel affinity tag for the capture and immobilization of coiled-coil proteins: application to the study of human β-cardiac myosin**

^1^Bipasha Barua, ^2^Robert C. Cail, ^2,3^Yale E. Goldman, ^2^E. Michael Ostap and ^1^Donald A. Winkelmann

^1^Department of Pathology and Laboratory Medicine, Robert Wood Johnson Medical School, Rutgers University, Piscataway, NJ 08854, ^2^Department of Physiology and Pennsylvania Muscle Institute, Perelman School of Medicine, University of Pennsylvania, Philadelphia, PA 19104, and ^3^Department of Pharmacology and Department of Molecular and Cellular Biology, University of California at Davis, Davis, CA 95616

Corresponding author:

Donald A. Winkelmann

Email: winkelma@rwjms.rutgers.edu

Department of Pathology and Laboratory Medicine, Robert Wood Johnson Medical School, Rutgers University, Piscataway, NJ 08854

**This document contains Supplementary Materials:**

- Figures S1-S4
- Movie S1

**A.** S2Tag and flanking sequences for myosin paralogs

41 42 43 44

Heptads *abcdefg abcdefg* *abcdefg abcdefg*

G. gallus FSk 1130 IEAERTS RAK**AEKH** **RADLSRE** LEEISER 1157

L. indicus IFM 1130 VEAERQA RGK**AEK**Q **RADL**A**RE** LEELGER 1157

H. sapien MYH1 1126 IEAERAS RAK**AEK**Q **R**S**DLSRE** LEEISER 1153

H. sapien MYH13 1126 IEAEHTL RAKI**EK**Q **R**S**DL**A**RE** LEEISER 1153

H. sapien βCARD 1122 LEAERTA RAKV**EK**L **R**S**DLSRE** LEEISER 1149

H. sapien αCARD 1124 LEAERTA RAKV**EK**L **R**S**DLSRE** LEEISER 1151

H. sapien SM2B 1124 LDSERAA RNK**AEK**Q KR**DL**GE**E** LEALKTE 1151

H. sapien NMIIA 1120 LESERAS RNK**AEK**Q KR**DL**GE**E** LEALKTE 1147

H. sapien NMIIB 1158 FESEKAS RNK**AEK**Q KR**DL**SE**E** LEALKTE 1185

H. sapien NMIIC 1152 LESERVS RTK**AEK**Q **R**R**DL**GE**E** LEALRGE 1179

H. sapien MYO5A 1225 LEYESLK RQEL**E**SE NKK**L**KN**E** LNELRKA 1252


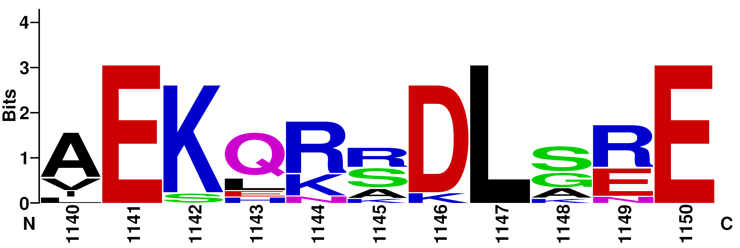


**B.** S2Tag is conserved in avian striated muscle myosin paralogs


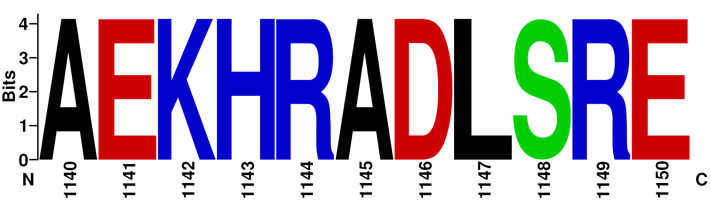


**Figure S1**. Myosin S2Tag sequence comparisons. **A**. Comparison of heptads 41-44 of the chicken fast skeletal muscle myosin S2 domain (G. gallus FSK) to L. indicus indirect flight muscle S2 segment used for the model in Figure 1 and to a cross section of human myosin paralogs: MYH1, fast skeletal myosin 1B; MYH13, fast extraocular myosin; β-cardiac (slow) myosin, MYH7; α-cardiac myosin, MYH6; MYH11, smooth muscle myosin II; MYH9, 10, and 11, non-muscle myosin IIA, IIB and IIC respectively; and unconventional myosin V, MYO5B. The consensus sequence for the epitope identifies conserved residues that position the epitope in the heptad repeats of the coiled-coil motif of the myosin S2 domain. **B**. S2Tag is conserved throughout the fast striated muscle paralogs of the avian clade as illustrated with this consensus for 94 distinct avian myosin sequences. Sequences were aligned with ClustalW and the WebLogo representation was composed online (weblogo.berkely.edu).


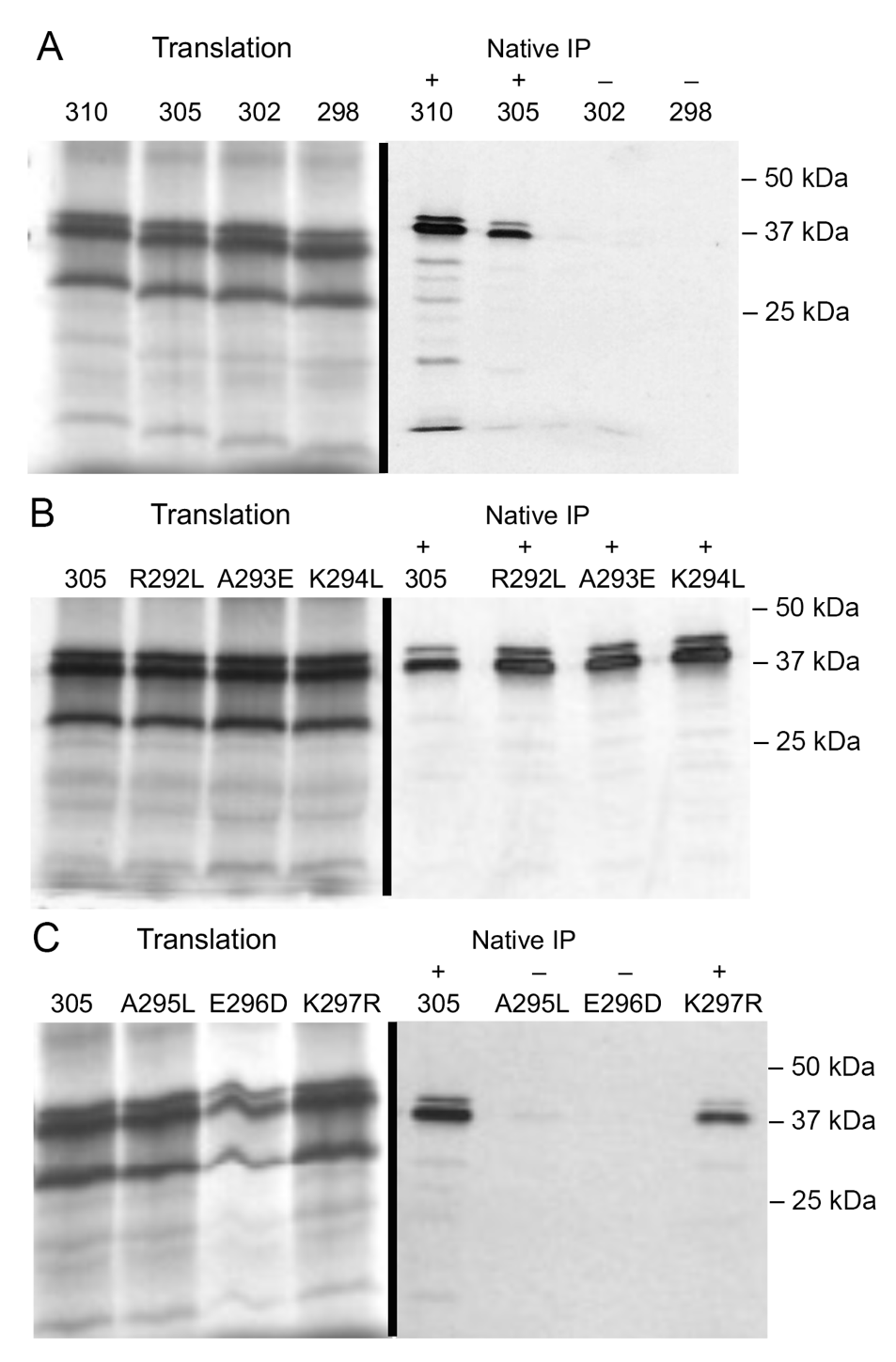


**Figure S2**. Expression and immunoprecipitation of S2 fragments with mAb 10F12.3. A series of S2 fragment truncations and point mutations were expressed in an *in vitro* transcription-translation assay and radiolabeled protein was assayed by immunoprecipitation with 10F12.3. **A.** Left panel is the autoradiograph of the SDS PAGE of the translation products for four S2 fragments truncated at positions ranging from 310 - 298 residues downstream from the start of the myosin rod (Fig. 1D). The right panel is the autoradiograph of the native immunoprecipitation (Native IP) with mAb 10F12.3 showing that the C-terminal limit of the epitope is 305 residues from the start of the S2 domain. **B, C.** A similar analysis probes the N-terminal limit of the epitope by site-directed mutagenesis of residues 292 - 297 of the 305 residue S2 fragment. Residues A295 and E296 are shown to be key to antibody recognition.

βcHMM

LC1

LC2


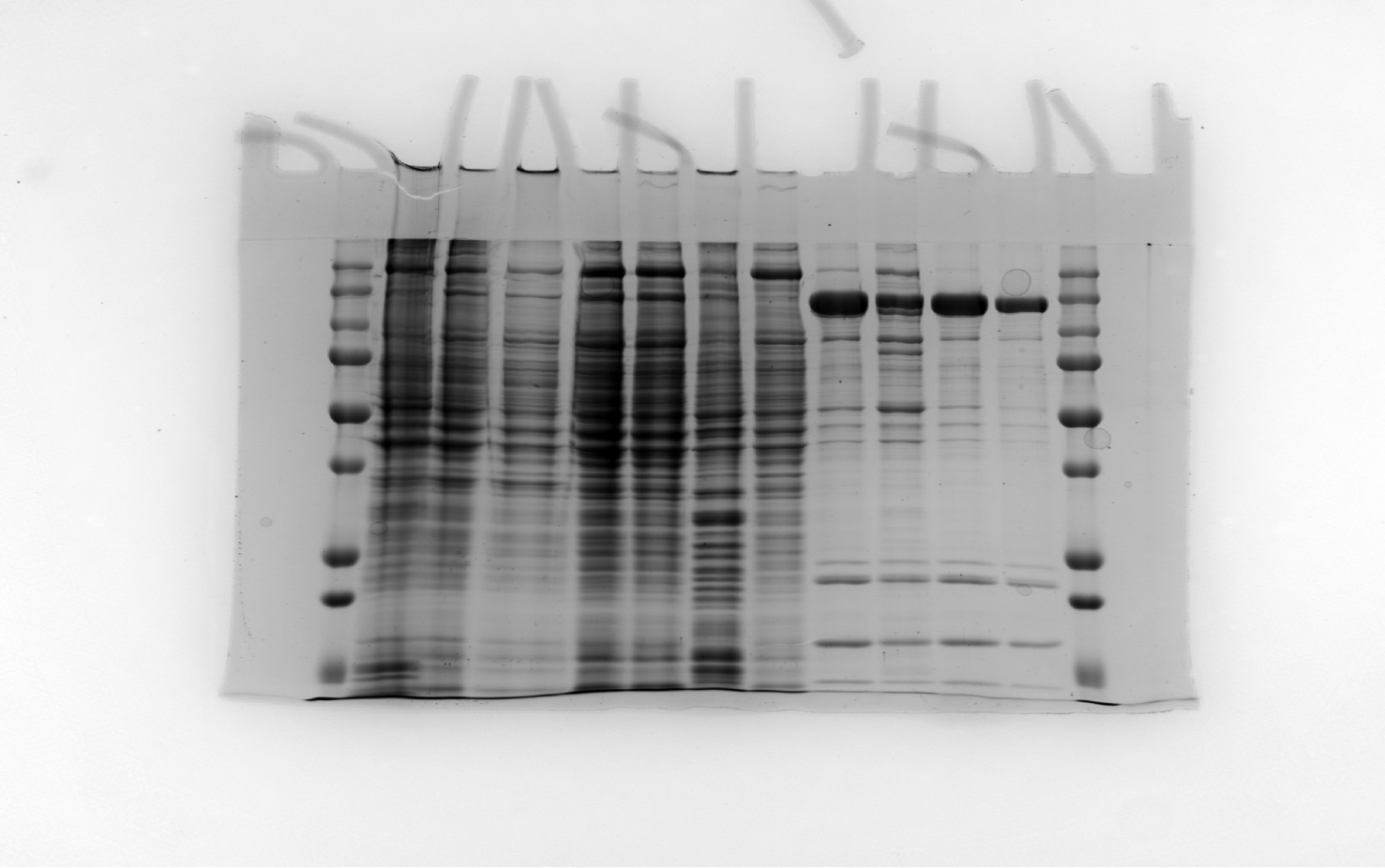

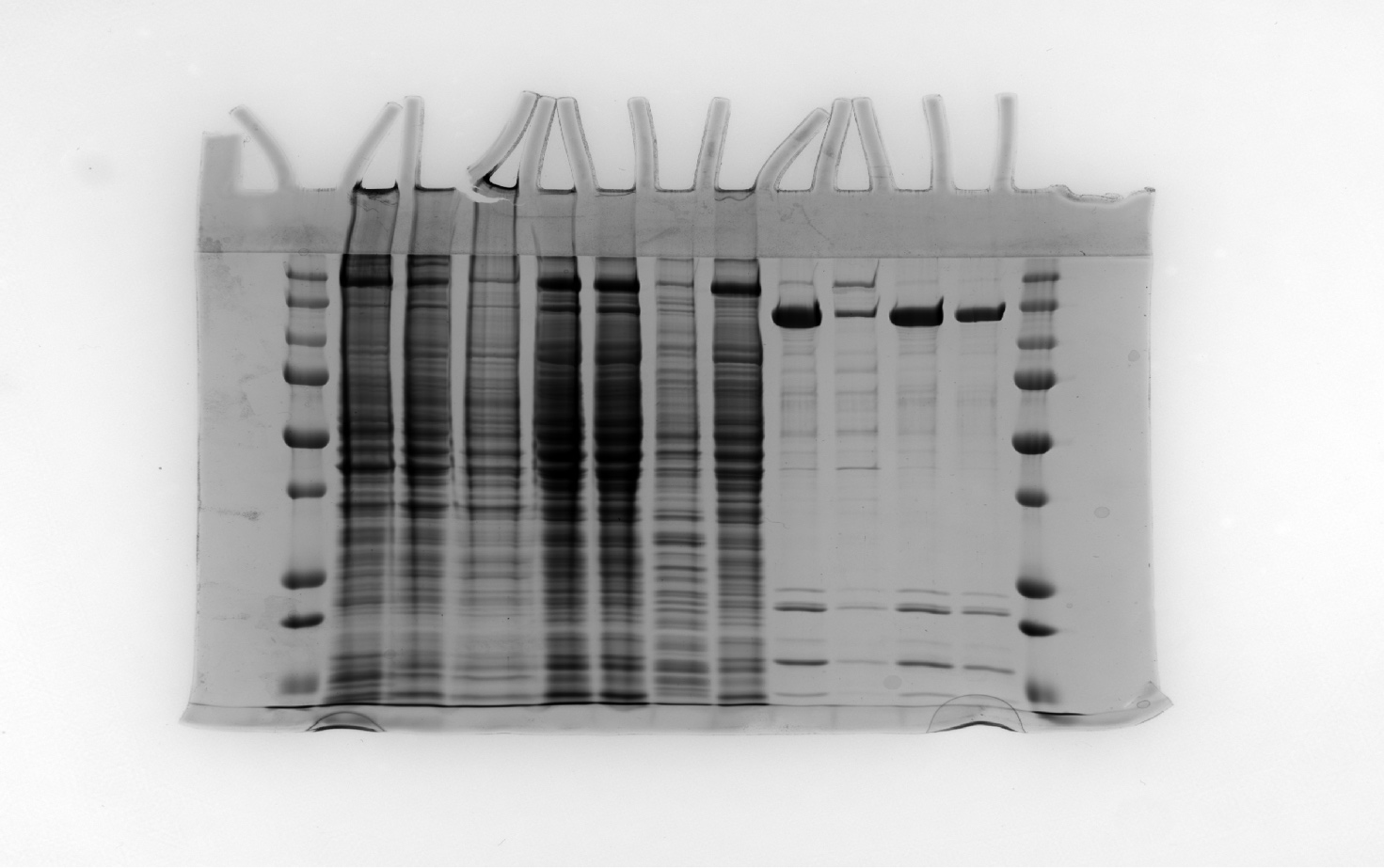


WT S532P

**Figure S3**. SDS polyacrylamide gel electrophoresis of typical purified WT- and S532P-cHMM2.0 protein samples used in this study. Proteins were expressed in C2C12 myotubes via adenovirus mediated delivery of a β-cHMM2.0 expression cassette (see Material and Methods). The purified β-cHMM2.0 has a 134 kDa heavy chain and associated essential and regulatory light chains. There is no detectable C2C12 myosin contamination in the samples.


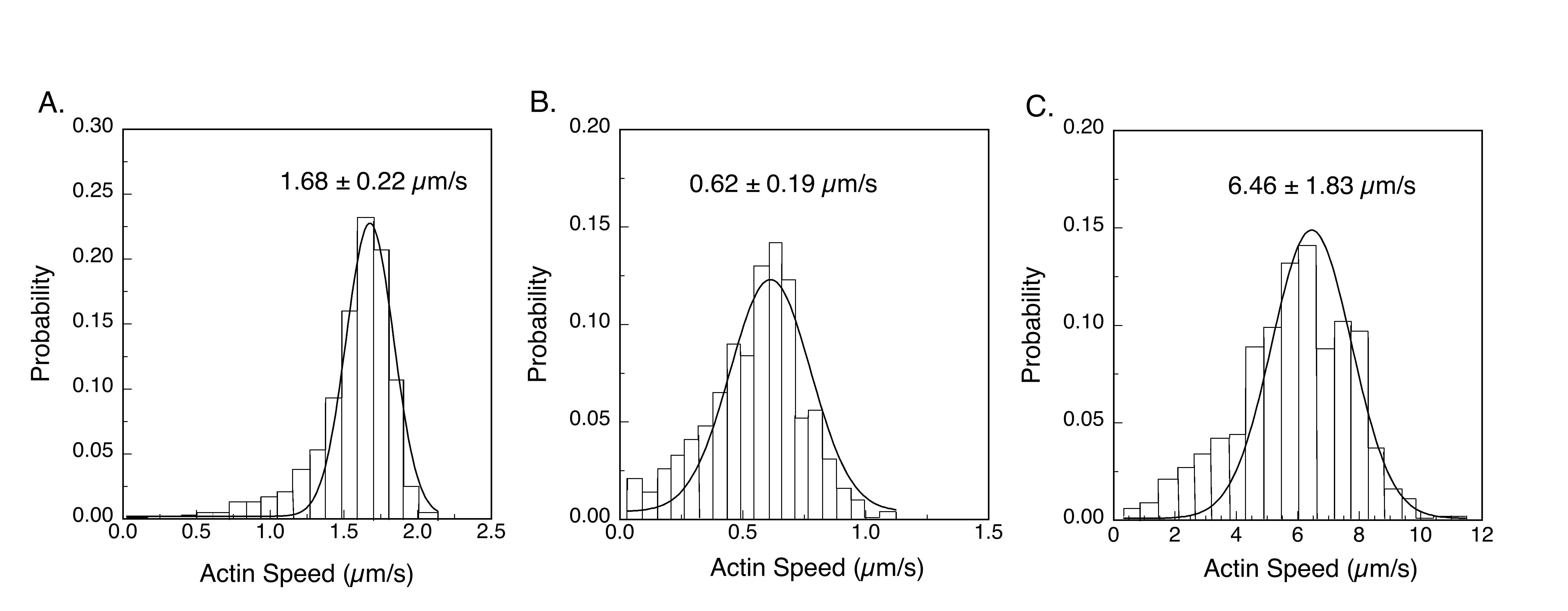


**Figure S4**. Probability distributions of actin filament speeds from motility assay movies. The data approximate a normal distribution with a mean gliding speed and standard deviation for: **A.** WT cHMM2.0, **B.** S532P cHMM2.0, and **C.** Sk-myosin. All proteins were bound to nitrocellulose coverslips coated with the mAb 10F12.3. Actin filament movement was captured in movies of 500 - 1,000 frames recorded at 5 - 15 frames/sec depending on filament speed and analyzed with a semi-automated filament tracking program. The trajectory of every filament with a lifetime of at least 10 frames is determined. The distance versus time plot for each filament is divided into segments of uniform velocity by the filament tracking programs. The velocity of the filament centroid moving on the trajectory, the filament length, the distance of continuous motion, and the duration of pauses are tabulated. The list of segments is sorted in descending order of the filament velocity. The minimum and maximum velocities are determined and divided into 20 bins between these limits. Defects that arise in the preparation of surfaces can result in occasional slowing and short pauses of the sliding movement. These events are not filtered out of the dataset. To minimize the influence of these events in determining the filament velocity, the binned velocity data are weighted based on the fraction of the total distance the filaments moved in each bin. The resulting velocity distribution reflects the mean velocity of smooth movement minimizing the effect of surface defects that slow and momentarily pin filaments. The weighted probability of the actin filament velocity for 1000 - 1500 segments are fit to a normal distribution and reported as a mean velocity and SD for each experimental condition. The data shown here were collected at a motor protein loading concentration of 10 μg/mL and assayed at 32°C.


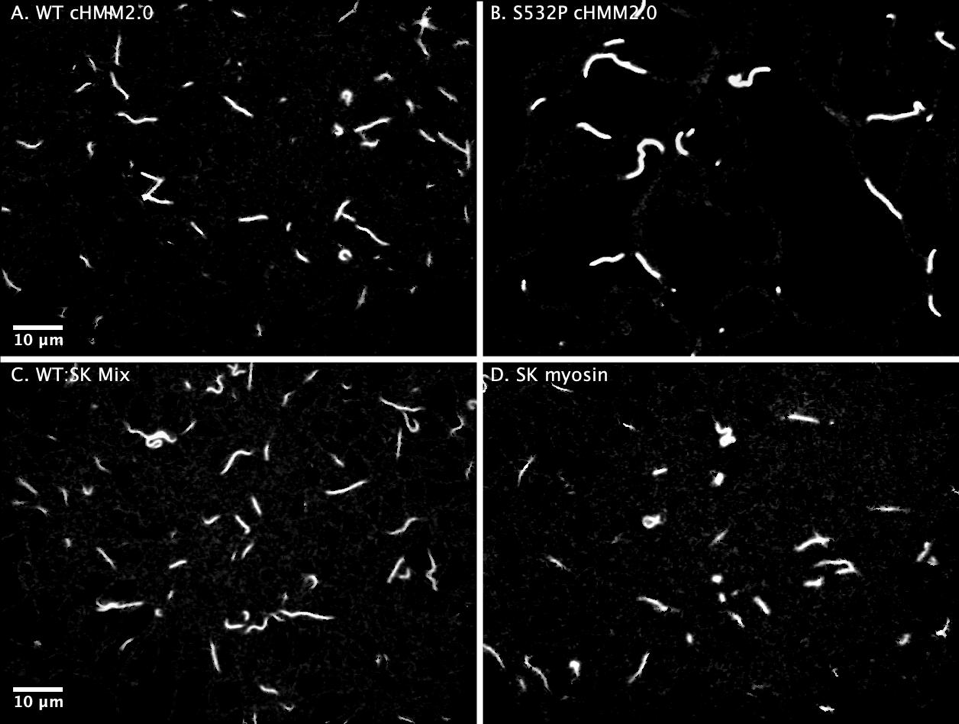


**Movie S1**. Motility assays for **A.** WT-cHMM2.0, **B.** S532P-cHMM2.0, **C.** WT:Sk-myosin mix (50:50), and **D.** Sk-myosin bound to nitrocellulose coverslips coated with mAb 10F12.3. Gliding actin filament movement was recorded at 5 fps for the slower motors: WT-cHMM2.0 (1.68 µm/s), and the S532P mutant (0.62 µm/s). The playback speed for these two panels is 3X faster than the recorded speed. The faster gliding speed of actin moving over the 50:50 mixture of WT-cHMM2.0 with Sk-myosin (3.0 µm/s), and Sk-myosin along (6.3 µm/s) were recorded and played back at 15 fps. All coverslips were prepared at 10 µg/mL motor protein loading. The 500-frame movies shown here were collected at 32°C and are representative of the data presented in Figures 2, 4, and S4 and tabulated in Table 1.
